# MicroRNA-5572 Is a Novel MicroRNA-Regulating *SLC30A3* in Sporadic Amyotrophic Lateral Sclerosis

**DOI:** 10.3390/ijms21124482

**Published:** 2020-06-24

**Authors:** Hisaka Kurita, Saori Yabe, Tomoyuki Ueda, Masatoshi Inden, Akiyoshi Kakita, Isao Hozumi

**Affiliations:** 1 Laboratory of Medical Therapeutics and Molecular Therapeutics, Gifu Pharmaceutical University, 1-25-4 Daigaku-nishi Gifu city, Gifu 501-1196, Japan; kurita@gifu-pu.ac.jp (H.K.); 155054@gifu-pu.ac.jp (S.Y.); 126014@gifu-pu.ac.jp (T.U.); inden@gifu-pu.ac.jp (M.I.); 2 Department of Pathology, Brain Research Institute, Niigata University, 1 Asahimachi, Chuo-ku, Niigata 951-8585, Japan; kakita@bri.niigata-u.ac.jp

**Keywords:** amyotrophic lateral sclerosis, microRNA, spinal cord, microarray

## Abstract

Amyotrophic lateral sclerosis (ALS) is a progressive degenerative disease caused by the loss of motor neurons. Although the pathogenesis of sporadic ALS (sALS) remains unclear, it has recently been suggested that disorders of microRNA (miRNA) may be involved in neurodegenerative conditions. The purpose of this study was to investigate miRNA levels in sALS and the target genes of miRNA. Microarray and real-time RT-PCR analyses revealed significantly-decreased levels of miR-139-5p and significantly increased levels of miR-5572 in the spinal cords of sALS patients compared with those in controls. We then focused on miR-5572, which has not been reported in ALS, and determined its target gene. By using TargetScan, we predicted *SLC30A3* as the candidate target gene of miR-5572. In a previous study, we found decreased *SLC30A3* levels in the spinal cords of sALS patients. We revealed that *SLC30A3* was regulated by miR-5572. Taken together, these results demonstrate that the level of novel miRNA miR-5572 is increased in sALS and that *SLC30A3* is one of the target genes regulated by miR-5572.

## 1. Introduction

Amyotrophic lateral sclerosis (ALS) is a progressive degenerative disease caused by the loss of motor neurons, which leads to muscular atrophy and weakness. The symptoms of ALS develop rapidly to typically cause death within 3–5 years owing to respiratory muscular paralysis. In Japan, the majority (approximately 95%) of ALS cases are sporadic of unknown cause (sALS), whereas the remaining 5% are familial (fALS). The causative genes in fALS have been reported to be *SOD1, TDP43, FUS*, and *C9orf72* [1]. Although the etiology of sALS remains unknown, environmental factors such as homeostasis of essential metals may play a pivotal role in the pathogenesis. Our previous studies showed increased levels of zinc (Zn) and copper (Cu) in the cerebral spinal fluid (CSF) of sALS patients [2], and decreased levels of the zinc transporters *SLC30A3* and SLC30A6 in the spinal cords of sALS patients compared with those of controls [3].

In drug treatments for ALS, few have successfully cured human ALS patients, although they have been effective in animal models such as *SOD1* mutant mice with fALS causative genes [4,5]. A better understanding of the molecular mechanism of sALS using autopsy samples is essential for novel drug development. In recent years, the relationship between epigenetics and the onset of neurodegenerative diseases has attracted considerable interest of both clinical and basic researchers. The epigenetic mechanisms underlying an etiology of ALS are important for clarifying the onset of sALS [6]. Epigenetics refers to mechanisms that change gene transcription without changing the DNA sequence, such as DNA methylation, histone modification, and microRNA (miRNA). MicroRNAs are short noncoding RNAs (about 20 bases) that bind to the 3′ untranslated region (3′-UTR) of target genes to suppress transcription and translation [7]. Studies of miRNAs in neurodegenerative diseases are expected to shed new light on molecular mechanisms and could identify biomarkers for diagnosis [8,9]. The purpose of this study was to determine novel miRNAs to help elucidate the mechanisms underlying the etiology of sALS.

## 2. Results

### 2.1. MiRNA Expression in Spinal Cords from Sporadic ALS Patients

**Figure 1:**
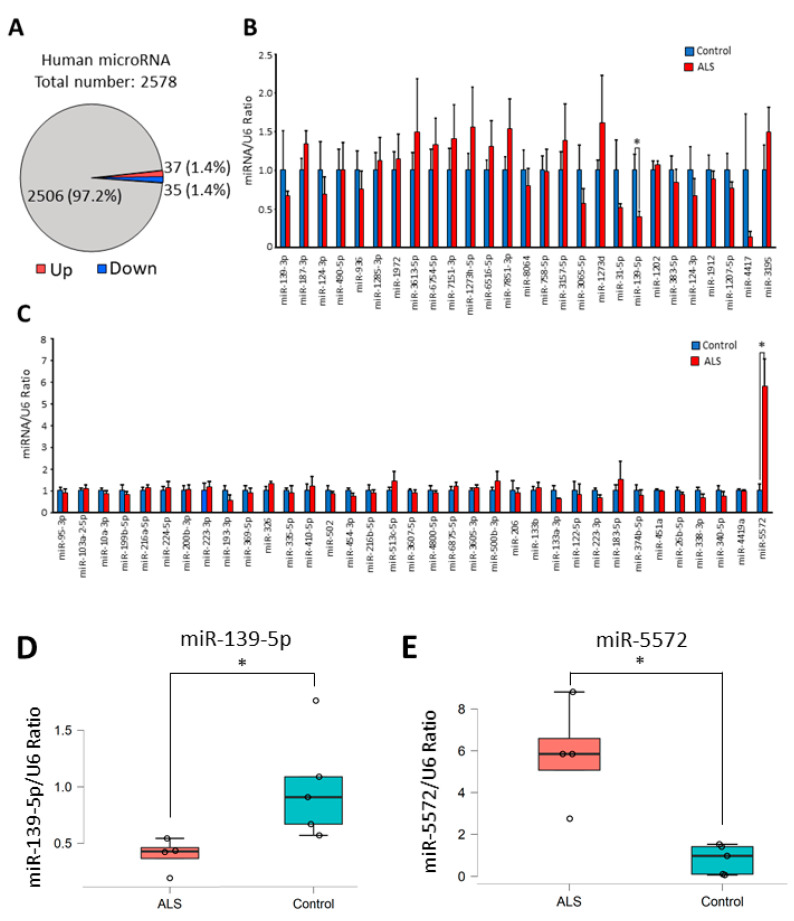
Global analysis of microRNA (miRNA) expression in sporadic amyotrophic lateral sclerosis (sALS) was performed using spinal cord samples from patients with sALS. (**A**) The number or ratio of altered miRNAs in sALS is presented. More than twofold changes in miRNA levels were calculated from the result of microarray analysis. (**B**) Decreases in miRNA levels in the microarray were confirmed by real-time RT-PCR analysis. All data are presented as mean ± standard error. Statistical significance between control (*n* = 5) and sALS (*n* = 4) group in each miRNA was determined by Student’s *t*-test (* *p* < 0.05). (**C**) Increases in miRNA levels in the microarray were confirmed by real-time RT-PCR analysis. All data are presented as mean ± standard error. Statistical significance between control (*n* = 5) and sALS (*n* = 4) group in each miRNA was determined by Student’s *t*-test (* *p* < 0.05). (**D**) Re-plotted individual data (control; *n* = 5 and sALS; *n* = 4) of miR-139-5p/U6 ratio in Figure 1B are presented as box and scatter plot. Statistical significance was determined by Student’s *t*-test (* *p* < 0.05). (**E**) Re-plotted individual data (control; *n* = 5 and sALS; *n* = 4) of miR-5572/U6 ratio in Figure 1B are presented as box and scatter plot. Statistical significance was determined by Student’s *t*-test (* *p* < 0.05).

Global analysis of miRNA expression in spinal cords from sALS patients was performed using a microarray. Raw data were validated by removing noise, which was data on low-signal spots. miRNAs showing twofold changes in their levels in sALS samples were identified: 37 miRNAs were upregulated and 35 miRNAs were downregulated (Figure 1A). All altered miRNAs in the ALS spinal cord samples are presented in the supplementary data (Supplementary Table S1). These results of the microarray analysis were further confirmed by real-time RT-PCR analysis. Among the miRNAs identified to show changes in their levels by the microarray analysis, miR-6076, miR-6721-5p, miR-448, miR-431-3p, miR-2276-3p, miR-3127-5p, miR-4638-5p, miR-5585-3p, miR-6800-5p, miR-6780b-5p, and miR-8063 could not be measured by real-time RT-PCR analysis, because suitable experimental conditions could not be determined. Real-time RT-PCR analysis confirmed that the level of miR-139-5p in sALS patients was significantly decreased compared with that in controls (Figure 1B,D), while the level of miR-5572 was significantly increased in sALS patients compared to controls (Figure 1C,E). 

### 2.2. SLC30A3 Expression in the Spinal Cords of Sporadic ALS Patients, and Determination of the Relationship between miR-5572 and SLC30A3

**Figure 2:**
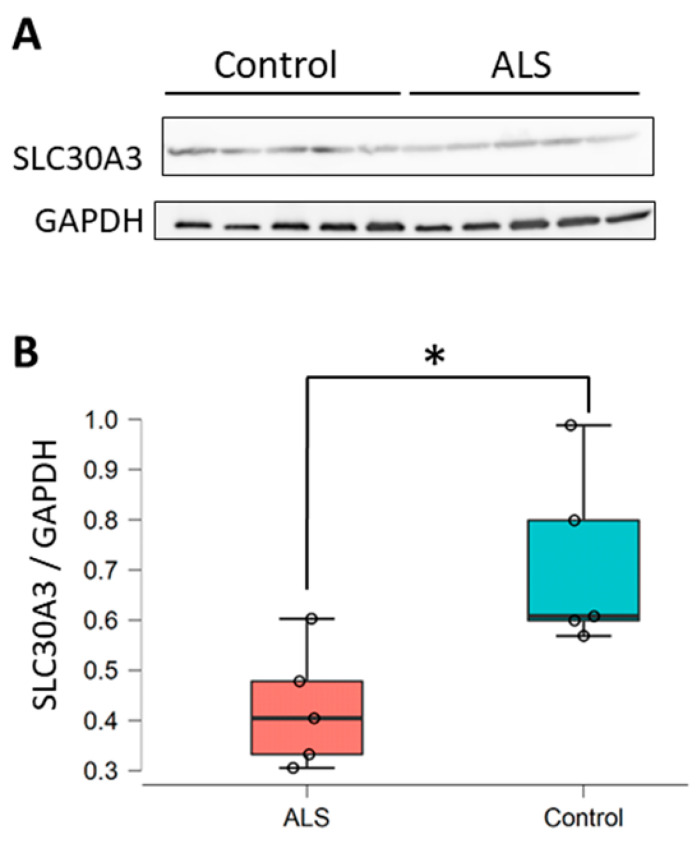
Determination of *SLC30A3* was performed using spinal cord samples from patients with sALS. (**A**) The levels of *SLC30A3* in the spinal cord were determined by Western blotting analysis. Samples from five patients in each experimental group (control; *n* = 5 and ALS; *n* = 5) were examined. (**B**) *SLC30A3* in the spinal cords of sALS patients was quantified (control; *n* = 5 and ALS; *n* = 5). Data are presented as box and scatter plot calculated from the band intensity of the Western blot. Statistical significance was determined by Student’s *t*-test (* *p* < 0.05).

Increased levels of miR-5572 and decreased levels of miR-139-5p were observed in the spinal cords of sALS patients. A change in miR-139-5p levels in the sera of ALS patients has been reported [10]. Alteration of miR-5572 has not been reported in ALS, and the functions or target genes of miR-5572 are unknown. We thus focused on this novel miRNA, miR-5572, and investigated its target genes. The candidate target genes of miR-5572 were predicted by using the web algorithm TargetScan. Increased levels of Zn and Cu in the CSF have been observed in ALS patients [2], and a disorder of Zn homeostasis could be an etiological factor for sALS. Thus, we focused on zinc transporters as the target genes. The zinc transporter genes *SLC30A3*, SLC30A7, SLC30A9, SLC39A7, SLC39A9, SLC39A11, and SLC39A14 were predicted as the targets of miR-5572 by using TargetScan. In addition, our previous study showed decreased *SLC30A3* levels in the spinal cords of sALS patients [3], and *SLC30A3* was chosen as a candidate target gene of miR-5572 in this study. The ALS spinal cord samples used in these two studies were collected from different patients. Thus, the *SLC30A3* levels were determined to assess whether these samples were comparable to the samples in the previous study. Decreased *SLC30A3* levels were confirmed in these samples (Figure 2), which corresponded to our previous study [3]. From this result, it is possible that the increase in the miR-5572 levels is related to the decrease in the *SLC30A3* levels in sALS patients. 

**Figure 3:**
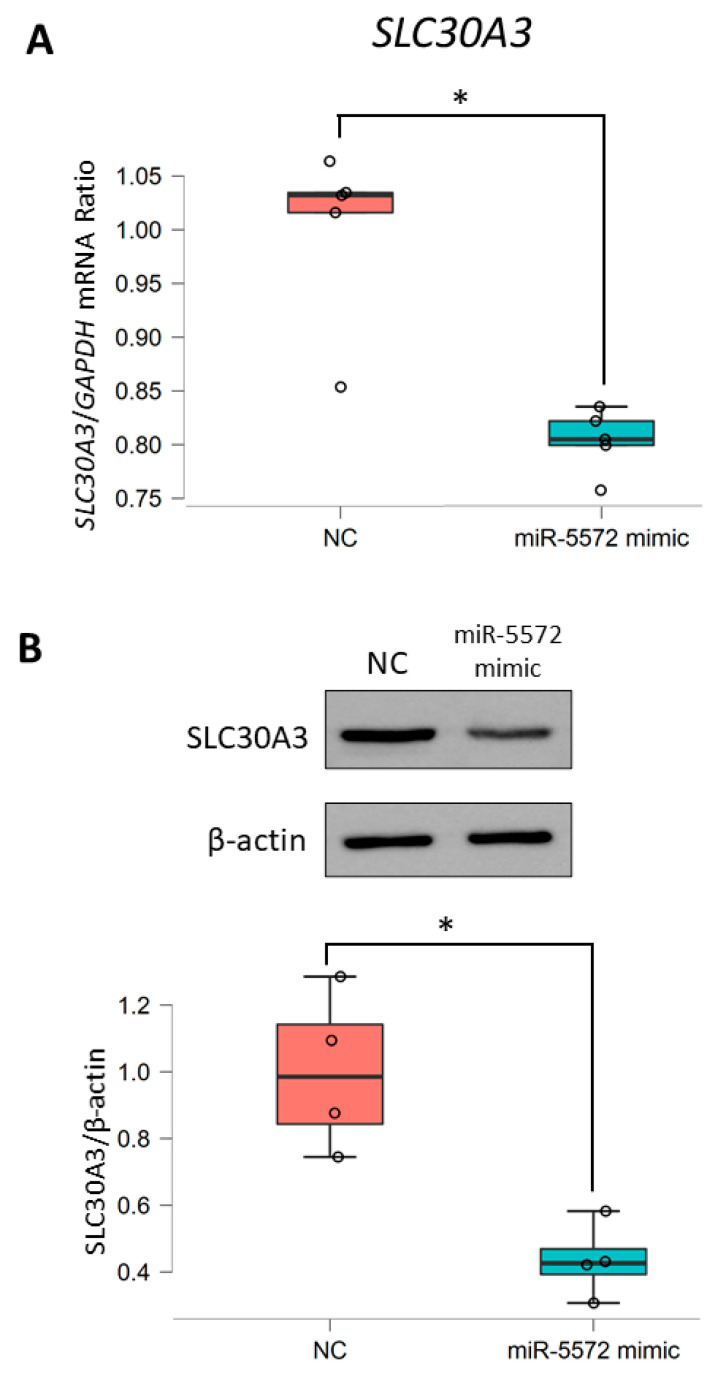
Determination of regulation of miR-5572 on *SLC30A3* expression. (**A**) The level of *SLC30A3* mRNA was determined in HEK293 cells transfected with negative control (NC) (*n* = 5) and miR-5572 mimic (*n* = 5) by real-time RT-PCR analysis. All data are presented as box and scatter plot. Statistical significance was determined by Student’s *t*-test (* *p* < 0.05). (**B**) The level of *SLC30A3* protein was determined in HEK293 cells transfected with NC (*n* = 4) or miR-5572 mimic (*n* = 4) by Western blotting analysis. All data are presented as box and scatter plot. Statistical significance was determined by Student’s *t*-test (* *p* < 0.05).

To investigate this possibility, we examined the transcriptional mechanism of *SLC30A3* regulated by miR-5572 using in vitro analysis. Transient overexpression of miR-5572 using a miRNA mimic was performed to determine the relationship between miR-5572 and *SLC30A3*. The levels of *SLC30A3* mRNA and protein expression in the miR-5572 mimic group were significantly decreased compared with those in the negative control (NC) group (Figure 3A–C). Thus, it is possible that miR-5572 regulates the level of *SLC30A3*. 

### 2.3. Determination of Effects of miR-5572 on 3′-UTR of SLC30A3

**Figure 4:**
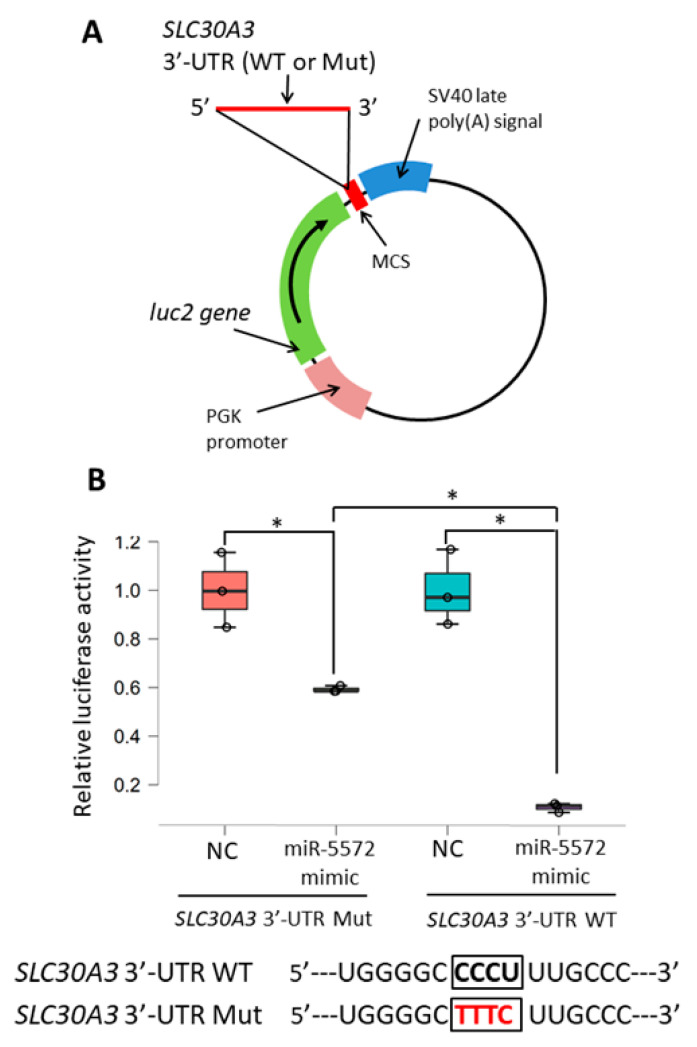
Reporter gene assay for the functional analysis of 3′-UTR regulated by miRNA. (**A**) The reporter construct contained the inserted DNA fragment of human *SLC30A3* 3′-UTR wild type (WT) or mutant (Mut) downstream of *luc2* in the pmirGLO Dual-Luciferase miRNA Target Expression Vector. (**B**) Reporter gene assay was performed using CHO cells co-transfected with NC or miR-5572 mimic, and the reporter vector cloned with WT 3′-UTR of *SLC30A3* or mutant 3′-UTR of *SLC30A3* (*n* = 3 in each experimental group). All data are presented as box and scatter plot. Statistical significance was determined by two-way ANOVA followed by post hoc Bonferroni’s test (* *p* < 0.05).

MiRNA binds to the 3′-UTR of the target gene to repress the level of the target gene. Here, a reporter system was used to examine the function of the 3′-UTR regulated by miRNA. Briefly, the 3′-UTR of the target gene was cloned in the reporter vector downstream of the luciferase gene. In this system, luciferase activity should be diminished if miRNA directly affects the cloned 3′-UTR in the reporter vector. Reporter vectors were constructed by cloning the 3′-UTR of *SLC30A3* (wild type, WT) or its mutant (Mut) downstream of the luciferase reporter gene (Figure 4A). Relative luciferase activity in WT was significantly decreased by the miR-5572 mimic in comparison with that in NC (Figure 4B). Relative luciferase activity was significantly increased by the miR-5572 mimic in the Mut group compared with that in the WT group, although the decrease in luciferase activity remained significant between NC and miR-5572 mimic in the Mut group (Figure 4B). MiR-5572 is thus considered to bind to the 3′-UTR of *SLC30A3* to suppress the transcriptional activity of *SLC30A3*. 

## 3. Discussion

The pathophysiological mechanisms of sALS have not yet been elucidated. In this study, we aimed to elucidate the role of miRNA in sALS. Decreased levels of miR-139-5p and increased levels of miR-5572 were found in the spinal cords of sALS patients. Our study revealed for the first time that miR-5572 directly regulates *SLC30A3* expression, and that increased levels of miR-5572 are considered to contribute to decreased levels of *SLC30A3* in sALS. 

In previous studies on miRNA using samples obtained from ALS patients, researchers attempted to mainly identify biomarkers. For instance, increased levels of miR-206 were found in the skeletal muscle, serum, and plasma of patients with sALS [11,12,13], and the level of miR-338-3p was increased in the spinal cord, CSF, plasma, serum, and leukocytes of ALS patients [14,15]. These miRNAs are possible biomarkers for sALS. Although miR-206 is related to skeletal muscle differentiation [16], miRNAs examined in previous studies using samples obtained from patients with ALS have not been explicitly linked to the etiology of sALS. MiR-139-5p has been reported to suppress tumor progression [17,18], and decreased levels of miR-139-5p in the serum of patients with ALS have recently been reported [10]. In this study, the levels of miR-139-5p were decreased in the spinal cords of sALS patients, which is consistent with the previous study. Although a change in serum level is not necessarily reflected in the central nervous system, serum miR-139-5p might be a useful biomarker that reflects the status quo in sALS. Although there are limitations to using human post-mortem tissues to infer molecular mechanisms about the onset of sALS, further evidence of miRNAs in the brain, spinal tissue, or blood samples would be required to elucidate the relationship between miRNA dynamics and the etiology of ALS. At the same time, appropriate in vivo and in vitro experimental models of ALS should be used to determine the molecular mechanisms reflected by the evidence from human tissue samples.

We also found that the levels of miR-5572 increased in the spinal cords of sALS patients. To the best of our knowledge, this is the first report showing the relationship between miR-5572 and ALS. This miRNA is present only in humans and its functions have not been clarified yet. *SLC30A3* was identified as a target gene of miR-5572, and the regulatory mechanism of miR-5572 for *SLC30A3* expression has been verified in this study. Increased levels of miR-5572 are considered to contribute to the decreased levels of *SLC30A3* in sALS. Interestingly, *SLC30A3* plays a protective role against cellular stress, including ER stress [19] and oxidative stress [20]. Thus, the regulation of *SLC30A3* expression driven by miR-5572 suggests the molecular mechanisms of ER stress response. 

sALS is considered to be caused by a combination of genetic and environmental factors [21]. Dyshomeostasis of essential metals, including Zn and Cu, and other cellular stresses are assumed to be pathogenic environmental factors. Indeed, Zn participates in various important biological activities in proteins, including enzymes and transcription factors. Although several other target genes aside from the zinc transporters are considered to be related to the development of sALS, *SLC30A3* regulated by miR-5572 is a susceptibility gene for sALS. In the future, we should determine what causes the decrease in the miR-5572 levels in sALS patients in a given environment.

In conclusion, this study has shown that the levels of novel miRNAs were altered in the spinal cords of sALS patients, and that *SLC30A3* is one of the target genes regulated by miR-5572. Decreased levels of *SLC30A3* in sALS are considered to be related to increased levels of miR-5572. These findings are very valuable for determining a part of the epigenetic mechanisms of sALS. 

## 4. Materials and Methods 

### 4.1. Subjects and Ethics

Spinal cord samples were obtained from five sALS patients and five controls with diseases other than neurodegenerative disorders and no spinal lesions. Clinical procedures were performed in accordance with the Declaration of Helsinki, and this study was approved by the Ethics Committee of Gifu Pharmaceutical University (Approval number 28-2, 7 July 2016) and Graduate School of Medicine of Niigata University (2321, 7 October 2015). This study was registered with the UMIN Clinical Trials Registry approved by the International Committee of Medical Journal Editors (UMIN000030101, 24 November 2017). 

### 4.2. Microarray Analysis for miRNA from ALS Samples

Total RNA, including miRNA, was isolated from the spinal cord samples obtained from sALS patients and controls using a miRNeasy Mini Kit (Qiagen, Hilden, Germany). The quality of total RNA (RNA integrity number > 7.0) was assessed using a 2100 Bioanalyzer (Agilent Technology, Santa Clara, CA, USA). For each experimental group, 1 μg of pooled total RNA was labeled with biotin using a FlashTagTM Biotin HSR RNA Labeling Kit (Thermo Fisher Scientific, Waltham, MA, USA). The labeled RNA was then hybridized to a GeneChip^®^ miRNA 4.0 Array (Affymetrix, Santa Clara, CA, USA) in a GeneChip^®^ Hybridization Oven 645 (Affymetrix). The microarray was washed using GeneChip^®^ Fluidics Station 450 (Affymetrix) and scanned with a GeneChip^®^ Scanner 3000 7G (Affymetrix). The resulting data were analyzed using an Affymetrix^®^ GeneChip^®^ Command Console 4.0 (Affymetrix). Low signal-to-noise data were eliminated from the raw data. MiRNAs with changes in their levels greater than twofold were selected, and the changes were confirmed by real-time RT-PCR analysis.

### 4.3. Quantitative RT-PCR Analysis of miRNA

Total RNA (150 ng), including miRNA, was subjected to a reverse transcription reaction using a Mir-X™ miRNA First-Strand Synthesis Kit (TAKARA, Kusatsu, Japan) to form complementary DNA (cDNA), following the manufacturer’s protocol. An aliquot of cDNA was then amplified by real-time RT-PCR using a miRNA-specific forward primer and the universal reverse primer. Real-time RT-PCR was performed using THUNDERBIRD^®^ SYBR^®^ qPCR Mix (TOYOBO, Osaka, Japan) and a StepOne Real-Time PCR System (Applied Biosystems, Foster City, CA, USA) under the following conditions: 95 °C/60 s × 1 cycle; 95 °C/15 s, and 60–64 °C/45 s, × 40 cycles. MiRNA-specific primers were set following the manufacturer’s instructions, and the miRQ 3′ primer supplied by the manufacturer was used for the PCR. The miRNA-specific primers used for real-time RT-PCR are listed in Table 1. For each sample, the amplified U6 cDNA was used as an internal control. Primers supplied by the manufacturer were used to determine the U6 level.

### 4.4. Cell Culture 

Human embryonic kidney (HEK293) cells or Chinese hamster ovary (CHO) cells were used in this study. HEK293 cells were cultured in Dulbecco’s Modified Eagle’s medium (Sigma, St. Louis, MO USA) supplemented with 10% fetal bovine serum (FBS) under 5% CO_2_ at 37 °C. CHO cells were cultured in Ham’s medium (Sigma) supplemented with 10% FBS under 5% CO_2_ at 37 °C.

### 4.5. MicroRNA Mimic Experiments

The miRNA overexpression by the miRNA mimic was performed using the AccuTarget^TM^ “human” miRNA mimic “hsa-miR-5572” [Accession: MIMAT0022260] (BIONEER, Daejeon, Korea) or AccuTarget^TM^ miRNA-mimic-negative control #1 (BIONEER). HEK293 cells were seeded at a density of 7.0 × 10^5^ cells/well in a 6-well multiplate and cultured for 24 h. The miR5572 mimic or NC miRNA mimic was transfected into HEK293 cells or CHO cells using Lipofectamine RNAiMax (Invitrogen, Carlsbad, CA, USA) in Opti-MEM (Invitrogen). Transfected cells were subjected to real-time RT-PCR and Western blotting 24 h post-transfection.

### 4.6. Quantitative RT-PCR for mRNA

Total RNA was isolated using Tripure Isolation Reagent (Sigma) following the manufacturer’s protocols. cDNA was prepared from 1 μg of total RNA with ReverTra Ace^®^ qPCR RT Master Mix (TOYOBO), following the manufacturer’s protocols. Real-time RT-PCR analysis was performed using THUNDERBIRD^®^ SYBR qPCR Mix (TOYOBO) and amplified using a StepOne Real-Time PCR System under the following conditions: 95 °C/60 s × 1 cycle; 95 °C/15 s, 60 °C/45 s, × 40 cycles. The primers used were as follows: *SLC30A3* forward, 5′-ACCATGTTGCCTCTGCACAC-3′; reverse, 5′-CATCTCCGGCTGATACTGCTC-3′; *GAPDH* forward, 5′-TGGTGAAGACGCCAGTGGA-3′; reverse, 5′-GCACCGTCAAGGCTGAGAAC-3′. All primer sets were designed using the Primer3 program. The amplification of *GAPDH* cDNA in the sample was used as an internal control for all reactions of PCR amplification.

### 4.7. Western Blotting

Treated cells were lysed in lysis buffer (150 mM NaCl, 10 mM Tris-HCl, 10% glycerol, 1% triton X-100, 1% NP-40, 1 mM EDTA, 10 μg/mL aprotinin, 10 μg/mL leupeptin, 0.1 mM PMSF). The protein concentration was determined using a Pierce BCA protein assay kit (Thermo Fisher Scientific). Next, 15 μg of protein underwent SDS-PAGE to separate the proteins at a certain molecular weight. The separated proteins in polyacrylamide gel were transferred to a polyvinylidene fluoride membrane in transfer buffer (0.3% Tris, 1.44% glycine, 20% methanol). The membrane was incubated in 5% skim milk (Nacalai Tesque, Kyoto, Japan) at room temperature for 60 min. After blocking the reaction, the membrane was incubated with a primary antibody (1:1000) dissolved in 3% skim milk solution (rabbit anti-*SLC30A3* antibody, Proteintech Group, Rosemont, IL, USA) and a mouse anti-β-actin antibody (Santa Cruz Biotechnology, Dallas, TX, USA) at 4 °C overnight. After the primary antibody reaction, the membrane was incubated with a secondary antibody (goat anti-rabbit antibody conjugated with horseradish peroxidase (HRP), Santa Cruz Biotechnology, 1:2500) and a goat anti-mouse HRP antibody conjugated with HRP (Santa Cruz Biotechnology, 1:2500). Next, the membrane was incubated in ECL prime (GE Healthcare, Chicago, IL, USA) to generate chemiluminescence from the HRP-conjugated antibodies. Chemiluminescence was detected using a LAS3000 mini (Fujifilm, Tokyo, Japan) and the band density was measured using ImageJ software (NIH, Bethesda, MD, USA).

### 4.8. Reporter Construct of SLC30A3 3′-UTR

The pUC57 plasmid cloned with human *SLC30A3* 3′-UTR (pUC57; *hSLC30A3*; 3′-UTR) was purchased from GenScript. The human *SLC30A3* 3′-UTR was amplified from pUC57; *hSLC30A3*; 3′-UTR plasmid using PrimeSTAR^®^ Max DNA polymerase (TAKARA) and a Veriti Thermal Cycler (Applied Biosystems). A restriction enzymatic site of *XhoI* or *XbaI* was added to each primer to amplify *SLC30A3* 3′-UTR as follows: XhoI-3′UTR-*hSLC30A3*-forward, 5′-GACCTCGAGGCCATGGCCCT-3′; 3′UTR-*hSLC30A3*-XbaI-reverse, 5′-GCATCTAGATGCAGTGAGAC-3′. The amplified DNA fragment and pmirGLO Dual-Luciferase miRNA Target Expression Vector (Promega, Madison, WI, USA) were digested with the *XhoI* and *XbaI* restriction enzymes (Thermo Fisher Sciences). The digested DNA fragment was then subcloned into the digested pmirGLO Dual-Luciferase miRNA Target Expression Vector. This plasmid was termed pmirGLO; *hSLC30A3*; 3′UTR. The mutant of human *SLC30A3* 3′UTR was added to pmirGLO; *hSLC30A3*; 3′UTR by inverse PCR with PrimeSTAR^®^ Max DNA polymerase. The primers for generating the mutant plasmid were as follows: (*hSLC30A3*-3′UTR-mut-forward: 5′-ACAAGCCCGCACTTTGTCCGTGTGT-3′; *hSLC30A3*-3′UTR-mut-reverse: 5′-TTGCCCCTGCATAGACAGAGCGAGG-3′. This mutant plasmid was termed pmiRGLO; *hSLC30A3*; 3′UTR-mut.

### 4.9. Luciferase Reporter Gene Assay

CHO cells in Opti-MEM (Invitrogen) at a concentration of 3.5 × 10^5^ cells/mL were co-transfected with pmirGLO; *hSLC30A3*; 3′UTR or pmiRGLO; *hSLC30A3*; 3′UTR-mut, and pSV-β-galactosidase control (Promega) vectors, and an NC mimic or a miR5572 mimic using Lipofectamine2000 (Invitrogen). At 24 h after transfection, transfected cells were lysed by adding 1× Passive Lysis Buffer (Promega) and incubated for 15 min at 4 °C. The supernatant was then collected. Next, 20 μL of the supernatant was used in the luciferase assay and 50 μL of the supernatant was used to measure β-galactosidase activity. For the luciferase assay, 100 μL of luciferin reagent (0.5 mM luciferin, 20 mM tricine, 1.1 mM MgCO_2_, 2.7 mM MgSO_4_, 33.3 mM DTT, 0.2 mg/mL coenzyme A, 0.53 mM ATP, 0.1 mM EDTA) was added to the supernatant and incubated for 10 min at 37 °C. For the β-galactosidase activity assay, 50 μL of ONPG reagent (1.33 mg/mL 2-nitrophenyl-β-d-galactopyranoside, 120 mM Na_2_HPO_4_, 80 mM NaH_2_PO_4_, 2 mM MgCl_2_, 100 mM 2-mercaptoethanol) was added to the supernatant and incubated for 15 min at 37 °C. The levels of luminescence and β-galactosidase activity were measured using a GloMax-Multi Detection System (Promega), and luciferase activity was normalized using β-galactosidase activity.

### 4.10. Statistical Analysis

All results are expressed as mean ± standard error or box and scatter plot. Statistical analysis was performed using IBM SPSS Statistics ver. 19.0 (IBM, Westchester, NY, USA) or StatView (Abacus, Baltimore, MD, USA). The level of statistical significance was set at *p* < 0.05. Figures of box and scatter plot were described using JASP ver. 0.12.2 (University of Amsterdam, Amsterdam, The Netherlands).

## 5. Conclusions

The levels of novel miRNAs were altered in sALS. As *SLC30A3* is one of the target genes regulated by miR-5572, the decreased levels of *SLC30A3* in sALS are considered to be due to the increased levels of miR-5572.

## Figures and Tables

**Table 1:** Primer list for miRNA expression in real time RT-PCR experiments.

miRNA-Specific Primers (5′ > 3′)	Target miRNA	miRNA-Specific Primers (5′ > 3′)	Target miRNA
TTCAACGGGTATTTATTGAGCA	miR-95-3p	AGGCAAGATGCTGGCATAGCT	miR-31-5p
AGCTTCTTTACAGTGCTGCCTTG	miR-103a-2-5p	TCTACAGTGCACGTGTCTCCAGT	miR-139-5p
CAAATTCGTATCTAGGGGAATA	miR-10a-3p	GTGCCAGCTGCAGTGGGGGAG	miR-1202
CCCAGTGTTTAGACTATCTGTTC	miR-199b-5p	AGATCAGAAGGTGATTGTGGCT	miR-383-5p
TAATCTCAGCTGGCAACTGTGA	miR-216a-5p	TAAGGCACGCGGTGAATGCC	miR-124-3p
CAAGTCACTAGTGGTTCCGTT	miR-224-5p	TACCCAGAGCATGCAGTGTGAA	miR-1912
TAATACTGCCTGGTAATGATGA	miR-200b-3p	TGGCAGGGAGGCTGGGAGGGG	miR-1207-5p
TGTCAGTTTGTCAAATACCCCA	miR-223-3p	GGTGGGCTTCCCGGAGGG	miR-4417
AACTGGCCTACAAAGTCCCAGT	miR-193-3p	CGCGCCGGGCCCGGGTT	miR-3195
TTATAAAGCAATGAGACTGATT	miR-369-5p	TTATAAAGCAATGAGACTGATT	miR-340-3p
CCTCTGGGCCCTTCCTCCAG	miR-326	TGAGGGAGGAGACTGCA	miR-4419a
CAAGAGCAATAACGAAAAATGT	miR-335-5p	GTTGGGGTGCAGGGGTCTGCT	miR-5572
AATATAACACAGATGGCCTGT	miR-410-3p	TGGAGACGCGGCCCTGTTGGAGT	miR-139-3p
ATCCTTGCTATCTGGGTGCTA	miR-502-5p	TCGTGTCTTGTGTTGCAGCCGG	miR-187-3p
TAGTGCAATATTGCTTATAGGGT	miR-454-3p	TAAGGCACGCGGTGAATGCC	miR-124-3p
AAATCTCTGCAGGCAAATGTGA	miR-216b-5p	CCATGGATCTCCAGGTGGGT	miR-490-5p
TTCTCAAGGAGGTGTCGTTTAT	miR-513c-5p	GATGGTTGACCAGAGAGCACAC	miR-758-5p
GCACCCAGGCAAGGATTCTG	miR-500b-3p	ACAGTAGAGGGAGGAATCGCAG	miR-936
CCTCCGTGTTACCTGTCCTCTAG	miR-3605-3p	TCTGGGCAACAAAGTGAGACCT	miR-1285-3p
GCATGTGATGAAGCAAATCAGT	miR-3607-5p	TCAGGCCAGGCACAGTGGCTCA	miR-1972
AGTGGACCGAGGAAGGAAGGA	miR-4800-5p	TTCAGCCAGGCTAGTGCAGTCT	miR-3157-5p
TGGAATGTAAGGAAGTGTGTGG	miR-206	TCAACAAAATCACTGATGCTGGA	miR-3065-5p
TTTGGTCCCCTTCAACCAGCTA	miR-133b	GAACCCATGAGGTTGAGGCTGCAGT	miR-1273d
TTTGGTCCCCTTCAACCAGCTG	miR-133a-3p	TGTTGTACTTTTTTTTTTGTTC	miR-3613-5p
TGGAGTGTGACAATGGTGTTTG	miR-122-5p	CCAGGGAGGCTGGTTTGGAGGA	miR-6754-5p
TGTCAGTTTGTCAAATACCCCA	miR-223-3p	CTACAGGCTGGAATGGGCTCA	miR-7151-3p
TATGGCACTGGTAGAATTCACT	miR-183-5p	CTGGGAGGTCAAGGCTGCAGT	miR-1273h-5p
AAACCGTTACCATTACTGAGTT	miR-451a	TTTGCAGTAACAGGTGTGAGCA	miR-6516-5p
ATATAATACAACCTGCTAAGTG	miR-374b-5p	TACCTGGGAGACTGAGGTTGGA	miR-7851-3p
TTCAAGTAATTCAGGATAGGT	miR-26b-5p	AGCACACTGAGCGAGCGGAC	miR-8064
TCCAGCATCAGTGATTTTGTTG	miR-338-3p		

## Supplementary Materials

Supplementary Materials can be found at https://www.mdpi.com/1422-0067/21/12/4482/s1. Table S1: Up-regulated miRNA in ALS. Table S2: Down-regulated miRNA in ALS.

## Abbreviations

ALS	amyotrophic lateral sclerosis
miRNA	microRNA
CSF	cerebral spinal fluid
UTR	untranslated region
