# Supplementary material for: MicroRNA-5572 Is a Novel MicroRNA-Regulating SLC30A3 in Sporadic Amyotrophic Lateral Sclerosis
Source: Int J Mol Sci. 2020 Jun 24;21(12):4482. doi: 10.3390/ijms21124482 (PMC7350020; doi:10.3390/ijms21124482)
Supplement: Supplementary file 1 [file ijms-21-04482-s001.pdf]

Supplemental Table 1. Up-regulated miRNA in ALS

Expression value {ALS}	Expression value {Control}	Ratio	Log2Ratio	Transcript ID(Array Design)	Alignments	Sequence
1464.853	180.954	8.0951678	3.017061	hsa-miR-206	chr6:52009199-52009220 (+)	UGGAAUGUAAGGAAGUGUGUGG
147.059	30.19697	4.8699919	2.2839194	hsa-miR-133b	chr6:52013786-52013807 (+)	UUUGGUCCCCUUCAACCAGCUA
50.76917	13.39411	3.7904101	1.922354	hsa-miR-10a-3p	chr17:46657226-46657247 (-)	CAAAUUCGUAUCUAGGGGAAUA
25.39761	7.049512	3.6027473	1.8490974	hsa-miR-183-3p	chr7:129414768-129414789 (-)	GUGAAUUACCGAAGGGCCAUAA
19.06265	5.306083	3.5926031	1.8450295	hsa-miR-340-5p	chr5:179442361-179442382 (-)	UUAUAAAGCAAUGAGACUGAUU
13.24187	3.909132	3.3874195	1.7601867	hsa-miR-95-3p	chr4:8007039-8007060 (-)	UUCAACGGGUAUUUAUUGAGCA
6.429382	1.911846	3.3629184	1.7497138	hsa-miR-502-5p	chrX:49779221-49779241 (+)	AUCCUUGCUAUCUGGGUGCUA
6.978383	2.075337	3.36253	1.7495472	hsa-miR-216a-5p	chr2:56216155-56216176 (-)	UAAUCUCAGCUGGCAACUGUGA
278.0903	88.29298	3.1496309	1.6551828	hsa-miR-133a-3p	chr18:19405673-19405694 (-) // chr20:61162177-61162198	UUUGGUCCCCUUCAACCAGCUG
44.49857	14.47254	3.0746897	1.6204408	hsa-miR-216b-5p	chr2:56227899-56227920 (-)	AAAUCUCUGCAGGCAAAUGUGA
26.23337	9.924257	2.6433586	1.4023722	hsa-miR-3607-5p	chr5:85916325-85916346 (+)	GCAUGUGAUGAAGCAAAUCAGU
28.36707	10.97481	2.5847436	1.3700212	hsa-miR-200b-3p	chr1:1102540-1102561 (+)	UAAUACUGCCUGGUAAUGAUGA
11.42488	4.437901	2.5743882	1.3642296	hsa-miR-103a-2-5p	chr20:3898151-3898173 (+)	AGCUUCUUUACAGUGCUGCCUUG
622.1795	245.616	2.5331391	1.3409263	hsa-miR-122-5p	chr18:56118320-56118341 (+)	UGGAGUGUGACAAUGGUGUUUG
168.2702	66.77013	2.5201419	1.3335049	hsa-miR-223-3p	chrX:65238779-65238800 (+)	UGUCAGUUUGUCAAAUACCCCA
8.818762	3.533532	2.4957357	1.3194652	hsa-miR-4419a	chr1:23384373-23384389 (-)	UGAGGGAGGAGACUGCA
15.44541	6.207881	2.4880326	1.3150054	hsa-miR-224-5p	chrX:151127103-151127123 (-)	CAAGUCACUAGUGGUUCCGUU
360.2406	146.262	2.4629815	1.3004058	hsa-miR-183-5p	chr7:129414807-129414828 (-)	UAUGGCACUGGUAGAAUUCACU
28.517	11.74655	2.4276915	1.2795851	hsa-miR-410-3p	chr14:101532298-101532318 (+)	AAUAUAACACAGAUGGCCUGU
37.57158	15.83509	2.3726787	1.2465167	hsa-miR-5572	chr15:80873520-80873540 (+)	GUUGGGGUGCAGGGGUCUGCU
14.72862	6.233734	2.3627283	1.2404538	hsa-miR-193a-3p	chr17:29887069-29887090 (+)	AACUGGCCUACAAAGUCCCAGU
1621.209	696.3143	2.3282719	1.2192595	hsa-miR-451a	chr17:27188421-27188442 (-)	AAACCGUUACCAUUACUGAGUU
28.15777	12.23685	2.3010636	1.2023009	hsa-miR-326	chr11:75046152-75046171 (-)	CCUCUGGGCCCUUCCUCCAG
49.12244	21.73684	2.2598703	1.17624	hsa-miR-3605-3p	chr1:33798006-33798028 (-)	CCUCCGUGUUACCUGUCCUCUAG
38.72617	17.44977	2.219294	1.1501008	hsa-miR-335-5p	chr7:130135967-130135989 (+)	UCAAGAGCAAUAACGAAAAAUGU
8.667975	3.916714	2.2130733	1.1460512	hsa-miR-513c-5p	chrX:146271271-146271292 (-)	UUCUCAAGGAGGUGUCGUUUAU
65.6864	29.75305	2.2077199	1.1425571	hsa-miR-500b-3p	chrX:49775330-49775349 (+)	GCACCCAGGCAAGGAUUCUG
48.16438	22.34892	2.15511	1.1077615	hsa-miR-454-3p	chr17:57215148-57215170 (-)	UAGUGCAAUAUUGCUUAUAGGGU
5.791406	2.700037	2.1449358	1.1009345	hsa-miR-369-5p	chr14:101531943-101531964 (+)	AGAUCGACCGUGUUAUAUUCGC
169.9535	80.70351	2.1058997	1.0744367	hsa-miR-374b-5p	chrX:73438422-73438443 (-)	AUAUAAUACAACCUGCUAAGUG
201.6522	98.00868	2.0574933	1.0408877	hsa-miR-26b-5p	chr2:219267380-219267400 (+)	UUCAAGUAAUUCAGGAUAGGU
107.9958	52.58881	2.053589	1.0381475	hsa-miR-338-3p	chr17:79099687-79099708 (-)	UCCAGCAUCAGUGAUUUUGUUG
8.147333	3.980638	2.0467405	1.0333282	hsa-miR-199b-5p	chr9:131007062-131007084 (-)	CCCAGUGUUUAGACUAUCUGUUC
15.73682	7.688725	2.0467399	1.0333278	hsa-miR-6875-5p	chr7:100465663-100465683 (+)	UGAGGGACCCAGGACAGGAGA
24.58124	12.11218	2.0294646	1.0210991	hsa-miR-4800-5p	chr4:2251854-2251874 (-)	AGUGGACCGAGGAAGGAAGGA
8.735964	4.328875	2.018068	1.0129748	hsa-miR-6721-5p	chr6:32137861-32137883 (-)	UGGGCAGGGGCUUAUUGUAGGAG
11.92312	5.9554	2.0020687	1.0014915	hsa-miR-6076	chr14:50433117-50433137 (+)	AGCAUGACAGAGGAGAGGUGG

Supplemental Table 2. Down-regulated miRNA in ALS

Expression value {ALS}	Expression value {Control}	Ratio	Log2Ratio	Transcript ID(Array Design	Alignments	Sequence
144.7387	299.3523	0.483506223	-1.048393636	hsa-miR-31-5p	chr9:21512157-21512177 (-)	AGGCAAGAUGCUGGCAUAGCU
570.2968	1208.692	0.471829713	-1.08366182	hsa-miR-139-5p	chr11:72326146-72326168 (-)	UCUACAGUGCACGUGUCUCCAGU
26.45185	67.11097	0.394150912	-1.343179984	hsa-miR-139-3p	chr11:72326110-72326132 (-)	UGGAGACGCGGCCCUGUUGGAGU
11.94795	42.72344	0.279657958	-1.838264709	hsa-miR-187-3p	chr18:33484798-33484819 (-)	UCGUGUCUUGUGUUGCAGCCGG
1246.263	2705.317	0.460671707	-1.1181891	hsa-miR-6800-5p	chr19:50335279-50335299 (+)	GUAGGUGACAGUCAGGGGCGG
67.27836	149.1604	0.451047061	-1.148650128	hsa-miR-1202	chr6:156267943-156267963 (+)	GUGCCAGCUGCAGUGGGGGAG
2.493657	8.13407	0.306569405	-1.705714369	hsa-miR-448	chrX:114058087-114058108 (+)	UUGCAUAUGUAGGAUGUCCCAU
10.12342	23.70608	0.427038971	-1.227560362	hsa-miR-431-3p	chr14:101347406-101347427 (+)	CAGGUCGUCUUGCAGGGCUUCU
8.517908	26.08374	0.326560072	-1.614579691	hsa-miR-490-5p	chr7:136587952-136587971 (+)	CCAUGGAUCUCCAGGUGGGU
10.15396	24.54206	0.41373707	-1.273213868	hsa-miR-758-5p	chr14:101492371-101492392 (+)	GAUGGUUGACCAGAGAGCACAC
6.563102	24.71136	0.265590481	-1.912724652	hsa-miR-936	chr10:105807909-105807930 (-)	ACAGUAGAGGGAGGAAUCGCAG
217.7099	486.0221	0.447942388	-1.158614903	hsa-miR-383-5p	chr8:14710992-14711013 (-)	AGAUCAGAAGGUGAUUGUGGCU
1427.934	3199.78	0.446260055	-1.164043418	hsa-miR-124-3p	chr20:61809904-61809923 (+) // chr8:9760911-9760930 (-) // chr8:65291767-6529178	UAAGGCACGCGGUGAAUGCC
14.89144	45.15194	0.329807313	-1.600304707	hsa-miR-1285-3p	chr2:70480065-70480086 (-) // chr7:91833341-91833362 (-)	UCUGGGCAACAAAGUGAGACCU
63.15896	149.1332	0.423507039	-1.239542145	hsa-miR-1912	chrX:113886064-113886085 (+)	UACCCAGAGCAUGCAGUGUGAA
23.10405	61.653	0.374743321	-1.416025328	hsa-miR-1972	chr16:15104186-15104207 (-) // chr16:70064296-70064317 (+)	UCAGGCCAGGCACAGUGGCUCA
23.77014	51.27015	0.463625326	-1.108968718	hsa-miR-2276-3p	chr13:24736608-24736629 (+)	UCUGCAAGUGUCAGAGGCGAGG
4.193446	9.672431	0.433546231	-1.205742254	hsa-miR-3127-5p	chr2:97464025-97464047 (+)	AUCAGGGCUUGUGGAAUGGGAAG
3.200103	6.885526	0.464757957	-1.105448528	hsa-miR-3157-5p	chr10:97824126-97824147 (-)	UUCAGCCAGGCUAGUGCAGUCU
7.050548	19.3974	0.363479023	-1.460055989	hsa-miR-3065-5p	chr17:79099686-79099708 (+)	UCAACAAAAUCACUGAUGCUGGA
651.9526	1549.789	0.420671846	-1.249232827	hsa-miR-1207-5p	chr8:129061405-129061425 (+)	UGGCAGGGAGGCUGGGAGGGG
13.55906	37.30355	0.363479079	-1.460055766	hsa-miR-1273d	chr1:10287785-10287809 (+)	GAACCCAUGAGGUUGAGGCUGCAGU
3.161259	21.94248	0.144070269	-2.795155448	hsa-miR-3613-5p	chr13:50570601-50570622 (-)	UGUUGUACUUUUUUUUUUGUUC
326.4088	780.5117	0.418198472	-1.257740306	hsa-miR-4417	chr1:5624142-5624159 (+)	GGUGGGCUUCCCGGAGGG
3.628775	7.878541	0.460589721	-1.118445881	hsa-miR-4638-5p	chr5:180649612-180649632 (-)	ACUCGGCUGCGGUGGACAAGU
4.930579	15.26289	0.323043604	-1.630199182	hsa-miR-5585-3p	chr1:32552587-32552608 (+)	CUGAAUAGCUGGGACUACAGGU
23.77014	64.77013	0.366992316	-1.446178239	hsa-miR-6754-5p	chr11:71184554-71184575 (+)	CCAGGGAGGCUGGUUUGGAGGA
99.22543	258.2499	0.38422253	-1.379985976	hsa-miR-3195	chr20:60639868-60639884 (+)	CGCGCCGGGCCCGGGUU
19.01959	38.38282	0.495523518	-1.012974564	hsa-miR-6780b-5p	chr6:43402285-43402307 (+)	UGGGGAAGGCUUGGCAGGGAAGA
6.721282	19.66167	0.341846954	-1.548577525	hsa-miR-7151-3p	chr10:69163109-69163129 (-)	CUACAGGCUGGAAUGGGCUCA
29.90039	72.75297	0.410985146	-1.282841842	hsa-miR-1273h-5p	chr16:24214469-24214489 (+)	CUGGGAGGUCAAGGCUGCAGU
9.113739	20.16938	0.451860146	-1.146051776	hsa-miR-6516-5p	chr17:75085510-75085531 (+)	UUUGCAGUAACAGGUGUGAGCA
8.183274	23.62799	0.346338135	-1.529746843	hsa-miR-7851-3p	chr12:42717542-42717563 (-)	UACCUGGGAGACUGAGGUUGGA
32.65362	66.59151	0.490357104	-1.028095316	hsa-miR-8063	chr15:37265032-37265053 (-)	UCAAAAUCAGGAGUCGGGGCUU
9.580845	21.8831	0.437819367	-1.19159232	hsa-miR-8064	chr3:52880539-52880558 (-)	AGCACACUGAGCGAGCGGAC
